# Disparities in Reported Testing for 1p/19q Codeletion in Oligodendroglioma and Oligoastrocytoma Patients: An Analysis of the National Cancer Database

**DOI:** 10.3389/fonc.2021.746844

**Published:** 2021-11-09

**Authors:** Jad Zreik, Panagiotis Kerezoudis, Mohammed Ali Alvi, Yagiz U. Yolcu, Sani H. Kizilbash

**Affiliations:** ^1^ College of Medicine, Central Michigan University, Mount Pleasant, MI, United States; ^2^ Department of Neurologic Surgery, Mayo Clinic, Rochester, MN, United States; ^3^ Department of Medical Oncology, Mayo Clinic, Rochester, MN, United States

**Keywords:** 1p/19q codeletion, molecular testing, oligodendroglioma, oligoastrocytoma, disparities, adjuvant treatment

## Abstract

**Purpose:**

A chromosomal 1p/19q codeletion was included as a required diagnostic component of oligodendrogliomas in the 2016 World Health Organization (WHO) classification of central nervous system tumors. We sought to evaluate disparities in reported testing for 1p/19q codeletion among oligodendroglioma and oligoastrocytoma patients before and after the guidelines.

**Methods:**

The National Cancer Database (NCDB) was queried for patients with histologically-confirmed WHO grade II/III oligodendroglioma or oligoastrocytoma from 2011-2017. Adjusted odds of having a reported 1p/19q codeletion test for patient- and hospital-level factors were calculated before (2011-2015) and after (2017) the guidelines. The adjusted likelihood of receiving adjuvant treatment (chemotherapy and/or radiotherapy) based on reported testing was also evaluated.

**Results:**

Overall, 6,404 patients were identified. The reported 1p/19q codeletion testing rate increased from 45.8% in 2011 to 59.8% in 2017. From 2011-2015, lack of insurance (OR 0.77; 95% CI 0.62-0.97;p=0.025), lower zip code-level educational attainment (OR 0.62; 95% CI 0.49-0.78;p<0.001), and Northeast (OR 0.68; 95% CI 0.57-0.82;p<0.001) or Southern (OR 0.62; 95% CI 0.49-0.79;p<0.001) facility geographic region were negatively associated with reported testing. In 2017, Black race (OR 0.49; 95% CI 0.26-0.91;p=0.024) and Northeast (OR 0.50; 95% CI 0.30-0.84;p=0.009) or Southern (OR 0.42; 95% CI 0.22-0.78;p=0.007) region were negatively associated with reported testing. Patients with a reported test were more likely to receive adjuvant treatment (OR 1.73; 95% CI 1.46-2.04;p<0.001).

**Conclusion:**

Despite the 2016 WHO guidelines, disparities in reported 1p/19q codeletion testing by geographic region persisted while new disparities in race/ethnicity were identified, which may influence oligodendroglioma and oligoastrocytoma patient management.

## Introduction

Chromosomal 1p/19q codeletion status plays an important role in tumor diagnosis for patients with a histological diagnosis of oligodendroglioma or oligoastrocytoma. As the characteristic molecular signature of oligodendrogliomas, 1p/19q codeletion has been associated with improved overall and progression-free survival (1, 2). Randomized clinical trials have identified this mutation as a marker of enhanced response to chemoradiotherapy in anaplastic oligodendrogliomas (3, 4). Additionally, an isocitrate dehydrogenase (IDH) mutation has been previously shown to occur in nearly all gliomas harboring a 1p/19q codeletion (5, 6). As a result of these associations, the 2016 WHO classification of CNS tumors included the presence of an IDH mutation and 1p/19q codeletion as required criteria for diagnosing an oligodendroglioma in WHO grade II and grade III diffuse gliomas (7).

A number of studies have described inequitable access to neuro-oncological care among glioma patients. Factors such as race, socioeconomic status, and geography have been previously shown to influence receipt of treatment, access to high-volume facilities, and overall survival (OS) (8–11). Despite the increasing emphasis on molecular diagnostics, it is unknown whether similar disparities exist in testing for 1p/19q codeletion. Analyzing whether past disparities have been maintained despite the 2016 WHO guidelines may also better inform targets for quality improvement initiatives. Therefore, we sought to evaluate the trends, disparities, and potential impact of a reported 1p/19q codeletion test among oligodendroglioma and oligoastrocytoma patients before and after implementation of the 2016 WHO guidelines.

## Methods and Materials

### Data Source and Patient Selection

The National Cancer Database (NCDB) was queried for this study. The NCDB, a joint program between the Commission on Cancer (CoC) of the American College of Surgeons and American Cancer Society, is a clinical oncology outcomes database used to evaluate trends in cancer care, establish benchmarks for participating hospitals, and serve as a basis for quality improvement (12). The registry captures approximately 70% of all newly diagnosed cancer cases in the United States from over 1,500 CoC-accredited facilities (13). Given that patient data in the registry is deidentified, this study was exempt from Institutional Review Board (IRB) approval.

Patients were identified using International Classification of Diseases for Oncology, third revision (ICD-O-3) codes indicating a histological diagnosis of Grade II or Grade III oligodendroglioma (9450, 9451) or mixed oligoastrocytoma (9382) in the CNS (ICD-O-3 topography codes: C70.1-C72.9). Identified patients were also adults (age ≥18 years) who had positive histologic diagnostic confirmation (based on microscopic tissue examination). Patients diagnosed from 2011-2017 were initially included for an analysis of trends in reported 1p/19q codeletion testing rates. Then, the cohort was stratified into groups diagnosed from 2011-2015 or 2017 in order to evaluate disparities before and after implementation of the 2016 WHO classification of CNS tumors. A diagnosis in 2011 was chosen as the early cutoff since the NCDB Participant User File (PUF) notes that reporting of 1p/19q codeletion is likely underrepresented in 2010, the variable’s first year of reporting (14). Cases with missing values for patient demographics, except for facility setting and geographic region, were excluded. Unknown facility setting and geographic region were not excluded in order to attenuate potential selection bias given that the NCDB suppresses these variables for patients aged <40 (14).

### Primary Outcome

Reported 1p/19q codeletion tests were identified using the *CS Site Specific Factor 5* (Chromosome 1p: Loss of Heterozygosity) and *CS Site Specific Factor 6* (Chromosome 19q: Loss of Heterozygosity) variables. Patients who were reported as testing positive or negative for loss of heterozygosity for both variables were identified as having a reported 1p/19q codeletion test. Those documented as “test not done (test not ordered or not performed)” or “not documented in patient record” for at least one of the variables were identified as having an unreported 1p/19q codeletion test. The remaining patients, indicated as “test ordered, results not in chart”, were excluded since it was unclear whether a 1p/19q codeletion test was ultimately performed.

### Patient Characteristics

Patient characteristics included patient demographics, tumor properties, and cancer-directed therapies administered during the initial course of treatment, as defined in the NCDB PUF and detailed in **Supplementary Table 1** (14). Tumor location was classified as supratentorial, infratentorial, or not otherwise specified (NOS) or other CNS according to the ICD-O-3 topography code for the primary site. Tumor size was dichotomized as <5cm or ≥5cm in accordance with the previous literature (15, 16). Extent of resection (EOR) was categorized as biopsy only, subtotal resection (STR), or gross total resection (GTR) according to the American College of Surgeons CoC Facility Oncology Registry Data System manual (17).

### Statistical Analysis

The unadjusted annual percentage of patients with a reported test for codeletion from 2011-2017 was calculated and plotted. The Cochran-Armitage test was performed to evaluate the presence of statistically significant temporal trends. The cohort was subsequently divided into two groups to be independently analyzed: patients diagnosed from 2011-2015 and patients diagnosed in 2017. For both groups, patient characteristics were summarized using frequencies with proportions for categorical variables or medians with interquartile ranges (IQRs) for continuous variables. Comparisons were made between those with reported and unreported 1p/19q codeletion tests using Pearson’s chi-squared test for categorical variables or the two-sample t-test for continuous variables. In order to elucidate predictors of a reported 1p/19q test, unadjusted and adjusted odds ratios were calculated.

After merging the two patient groups, the adjusted odds of a reported 1p/19q codeletion test in 2017 versus 2011-2015 was calculated for all patients and for each patient subgroup. Finally, the unadjusted and adjusted odds of receiving adjuvant treatment based on reported testing for 1p/19q codeletion were evaluated. An interaction analysis between EOR and reported testing revealed a statistically significant interaction term for both receipt of chemotherapy (p=0.038) and receipt of radiotherapy (p=0.017). Therefore, for this analysis, adjuvant treatment was dichotomized as receipt of any adjuvant treatment (chemotherapy and/or radiotherapy) or no adjuvant treatment (neither chemotherapy nor radiotherapy). Given that the significant interactions occurred specifically with a GTR, the EOR variable was also dichotomized as a GTR or other resection.

Unadjusted and adjusted odds ratios were calculated using univariate and multivariable logistic regression, respectively. For all regression analyses, univariate logistic regression was initially performed, then variables with p<0.10 were included in the subsequent multivariable logistic regression model. Collinearity between covariates in the multivariable models was assessed using the variance inflation factor. Analyses were performed using R version 3.6.1 (18). P-values were two-sided and values <0.05 were considered statistically significant.

## Results

### Trends in Testing

**Figure 1:**
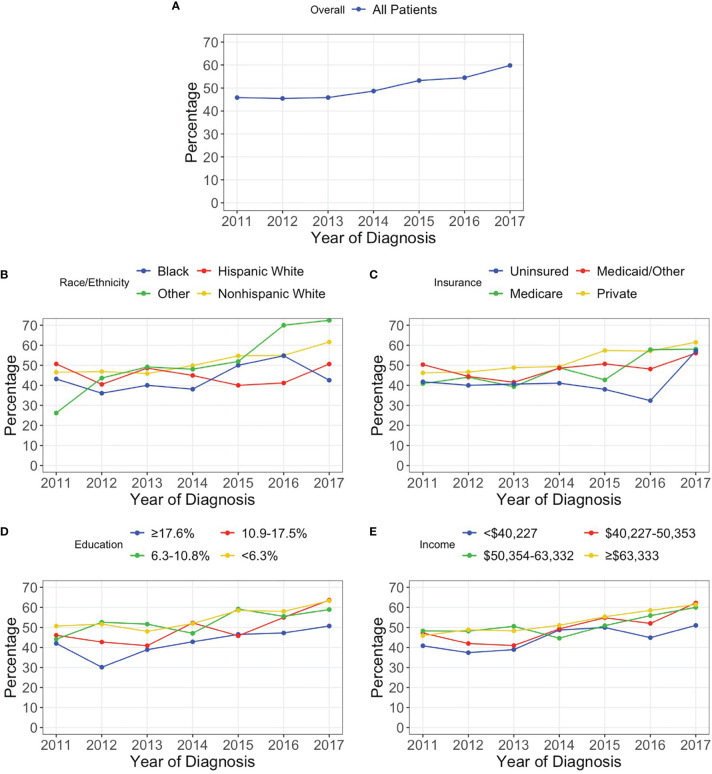
Percentage of patients with a reported 1p/19q codeletion test for **(A)** all patients and stratified by **(B)** race/ethnicity, **(C)** insurance status, **(D)** percentage of adults without a high school degree in patient’s zip code, and **(E)** median household income in patient’s zip code.

A total 6,404 patients were included in the analysis following exclusions. From 2011 to 2017, the percentage of patients in the NCDB with a reported test for 1p/19q codeletion increased from 45.8% to 59.8% (p<0.001). The Cochran-Armitage test also identified a statistically significant increasing trend for most patient subgroups. However, Hispanic White (p=0.922), Black (p=0.218), Medicaid/Other (p=0.154), and uninsured (p=0.462) patients did not experience a significant increase in reported codeletion testing over the study period (**Figure 1**).

### Disparities Before 2016 WHO Guidelines

A total of 4,931 patients diagnosed from 2011-2015 were identified; of which, 47.6% (n=2,349) had a reported 1p/19q codeletion test while 52.4% (n=2,582) did not. The median age was 44 years (IQR: 34-55) with 56.0% (n=2,763) being male. Non-Hispanic White, Hispanic White, Black, and other race/ethnicity patients had reported codeletion tests in 48.5%, 44.9%, 40.7%, and 44.7% of cases, respectively (p=0.034). Privately insured, Medicare, Medicaid/Other, and uninsured patients had reported testing rates of 49.4%, 43.0%, 47.3%, and 40.4%, respectively (p<0.001). Patients residing in a zip code in the top, second, third, and bottom quartiles of educational attainment had reported testing rates of 51.9%, 50.6%, 45.5%, and 39.95, respectively (p<0.001). Based on geographic region, a codeletion test was reported in 41.9%, 52.4%, 37.5%, and 55.3% of patients at Northeastern, Midwestern, Southern, and Western facilities, respectively (p<0.001). Significant differences in the rates of reported codeletion tests were also identified based on EOR (p<0.001) and receipt of adjuvant treatment (p<0.001) (**Table 1**).

**Table 1:** Characteristics of patients with and without a reported 1p/19q codeletion test diagnosed from 2011-2015. Frequencies and proportions are row-based.

Variable	Unreported 1p/19q test N = 2,582	Reported 1p/19q test N = 2,349	P-value
Age, median (IQR)	44 (34-56)	44 (33-54)	**0.004**
Sex			0.256
Male (n=2,763)	1427 (51.6%)	1336 (48.4%)	
Female (n=2,168)	1155 (53.3%)	1013 (46.7%)	
Race/Ethnicity			**0.034**
Non-Hispanic White (4,033)	2075 (51.5%)	1958 (48.5%)	
Hispanic White (n=356)	196 (55.1%)	160 (44.9%)	
Black (n=280)	166 (59.3%)	114 (40.7%)	
Other (n=262)	145 (55.3%)	117 (44.7%)	
Insurance			**<0.001**
Private (n=3,233)	1635 (50.6%)	1598 (49.4%)	
Medicare (n=609)	347 (57.0%)	262 (43.0%)	
Medicaid/Other (n=713)	376 (52.7%)	337 (47.3%)	
Uninsured (n=376)	224 (59.6%)	152 (40.4%)	
Median household income in zip code			**0.010**
<$40,227 (n=797)	455 (57.1%)	342 (42.9%)	
$40,227-50,353 (n=1,033)	553 (53.5%)	480 (46.5%)	
$50,354-63,332 (n=1,182)	609 (51.5%)	573 (48.5%)	
≥$63,333 (n=1,919)	965 (50.3%)	954 (49.7%)	
Adults without high school degree in zip code			**<0.001**
≥17.6% (n=972)	584 (60.1%)	388 (39.9%)	
10.9%-17.5% (n=1,177)	641 (54.5%)	536 (45.5%)	
6.3%-10.8% (n=1,434)	709 (49.4%)	725 (50.6%)	
<6.3% (n=1,348)	648 (48.1%)	700 (51.9%)	
Charlson-Deyo Comorbidity Score			0.552
0 (n=4,092)	2140 (52.3%)	1952 (47.7%)	
1 (n=569)	307 (54.0%)	262 (46.0%)	
2+ (n=270)	135 (50.0%)	135 (50.0%)	
Residential Region			0.472
Metropolitan (n=4,159)	2183 (52.5%)	1976 (47.5%)	
Urban (n=695)	364 (52.4%)	331 (47.6%)	
Rural (n=77)	35 (45.5%)	42 (54.5%)	
Geographic Region			**<0.001**
Northeast (n=1,123)	653 (58.1%)	470 (41.9%)	
Midwest (n=832)	396 (47.6%)	436 (52.4%)	
South (n=451)	282 (62.5%)	169 (37.5%)	
West (n=604)	270 (44.7%)	334 (55.3%)	
Unknown (n=1,921)	981 (51.1%)	940 (48.9%)	
Facility Setting			0.113
Academic (n=1,734)	902 (52.0%)	832 (48.0%)	
Non-academic (n=1,276)	699 (54.8%)	577 (45.2%)	
Unknown	981 (51.1%)	940 (48.9%)	
Distance travelled (miles), median (IQR)	14.8 (6.2-36.4)	17.5 (7.2-42.9)	0.368
1p/19q status			NA
Co-deleted	NA	1464	
Not co-deleted	NA	885	
Histology			**0.005**
Oligodendroglioma (n=3,253)	1657 (50.9%)	1596 (49.1%)	
Oligoastrocytoma (n=1,678)	925 (55.1%)	753 (44.9%)	
WHO grade			0.224
2 (n=2,859)	1476 (51.6%)	1106 (53.4%)	
3 (n=2,072)	1383 (48.4%)	966 (46.6%)	
Tumor location			**0.033**
Supratentorial (n=4,707)	2446 (52.0%)	2261 (48.0%)	
Infratentorial (n=68)	43 (63.2%)	25 (36.8%)	
NOS or other CNS (n=156)	93 (59.6%)	63 (40.4%)	
Tumor size			**0.002**
<5 cm (n=1,934)	996 (51.5%)	938 (48.5%)	
≥5 cm (n=1,690)	848 (50.2%)	842 (49.8%)	
Unknown (n=1,307)	738 (56.5%)	569 (43.5%)	
Extent of resection			**<0.001**
Biopsy only (n=498)	305 (61.2%)	193 (38.8%)	
STR (n=2,471)	1309 (53.0%)	1162 (47.0%)	
GTR (n=1,929)	946 (49.0%)	983 (51.0%)	
Unknown (n=33)	22 (66.7%)	11 (33.3%)	
Chemotherapy			**<0.001**
Yes (n=2,459)	1182 (48.1%)	1277 (51.9%)	
No (n=2,280)	1277 (56.0%)	1003 (44.0%)	
Unknown (n=192)	123 (64.1%)	69 (35.9%)	
Radiotherapy			0.070
Yes (n=2,389)	1218 (51.0%)	1171 (49.0%)	
No (n=2,492)	1335 (53.6%)	1157 (46.4%)	
Unknown (n=50)	29 (58.0%)	21 (42.0%)	
Adjuvant treatment			**<0.001**
Chemotherapy alone (n=578)	244 (42.2%)	334 (57.8%)	
Radiotherapy alone (n=476)	260 (54.6%)	216 (45.4%)	
Chemotherapy+Radiotherapy (n=1,874)	936 (49.9%)	938 (50.1%)	
Neither (n=1,783)	1005 (56.4%)	778 (43.6%)	
Unknown (n=220)	137 (62.3%)	83 (37.7%)	

Bold values indicate statistical significance (p < 0.05).

NA, not applicable.

On univariate analysis, Black (OR 0.73; 95% CI 0.57-0.93; p=0.011) and Medicare (OR 0.77; 95% CI 0.65-0.92; p=0.004) patients were significantly less likely to have a reported 1p/19q codeletion test; however, this significance was not retained after inclusion in the multivariable model. On multivariable analysis, factors including uninsured status (OR 0.77; CI 0.62-0.97; p=0.025), educational attainment in the third (OR 0.76; 95% CI 0.63-0.93; p=0.006) or bottom (OR 0.62; 95% CI 0.49-0.78; p<0.001) quartile, and hospital location in the Northeast (OR 0.68; 95% CI 0.57-0.82; p<0.001) and South (OR 0.62; 95% CI 0.49-0.79; p<0.001) compared to the Midwest were negatively associated with a reported 1p/19q codeletion test. Tumors with oligoastrocytoma histology were also less likely to have a reported codeletion test (OR 0.83; 95% CI 0.73-0.93; p=0.002) (**Table 2**).

**Table 2:** Univariate and multivariable logistic regression evaluating factors associated with reporting of a 1p/19q codeletion test for patients diagnosed from 2011-2015.

Variable	Reference	Univariate		Multivariable	
		OR (95% CI)	P-value	OR (95% CI)	P-value
Age	Continuous	0.99 (0.99-1.00)	**0.004**	0.99 (0.98-1.00)	**0.026**
Race/Ethnicity	Non-Hispanic White				
Hispanic White		0.87 (0.70-1.08)	0.192	0.99 (0.78-1.24)	0.906
Black		0.73 (0.57-0.93)	**0.011**	0.82 (0.64-1.06)	0.138
Other		0.86 (0.66-1.10)	0.222	0.85 (0.66-1.10)	0.231
Insurance	Private				
Medicare		0.77 (0.65-0.92)	**0.004**	0.93 (0.76-1.15)	0.527
Medicaid/Other		0.92 (0.78-1.08)	0.300	0.98 (0.83-1.16)	0.795
Uninsured		0.69 (0.56-0.86)	**<0.001**	0.77 (0.62-0.97)	**0.025**
Residential region	Metropolitan				
Urban		1.00 (0.86-1.18)	0.955	–	–
Rural		1.33 (0.84-2.09)	0.222	–	–
Charlson-Deyo Comorbidity score	0				
1		0.94 (0.78-1.12)	0.458	–	–
2+		1.10 (0.86-1.40)	0.464	–	–
Adults without high school degree in zip code	<6.3%				
6.3%-10.8%		0.95 (0.82-1.10)	0.470	0.94 (0.80-1.10)	0.425
10.9%-17.5%		0.77 (0.66-0.91)	**0.001**	0.76 (0.63-0.93)	**0.006**
≥17.6%		0.62 (0.52-0.73)	**<0.001**	0.62 (0.49-0.78)	**<0.001**
Median household income in zip code	<$40,227				
$40,227-50,353		1.15 (0.96-1.39)	0.130	0.97 (0.79-1.18)	0.742
$50,354-63,332		1.25 (1.04-1.50)	**0.015**	0.92 (0.75-1.13)	0.431
≥$63,333		1.32 (1.11-1.55)	**0.001**	0.88 (0.71-1.10)	0.274
Geographic region	Midwest				
Northeast		0.65 (0.55-0.78)	**<0.001**	0.68 (0.57-0.82)	**<0.001**
South		0.54 (0.43-0.69)	**<0.001**	0.62 (0.49-0.79)	**<0.001**
West		1.12 (0.91-1.39)	0.278	1.19 (0.96-1.48)	0.112
Unknown		0.87 (0.74-1.02)	0.094	0.77 (0.61-0.97)	**0.026**
Facility setting	Academic				
Nonacademic		0.89 (0.77-1.03)	0.133	**-**	**-**
Unknown		1.04 (0.91-1.18)	0.566	–	–
WHO grade	Grade II				
Grade III		0.93 (0.83-1.04)	0.224	–	–
Histology	Oligodendroglioma				
Oligoastrocytoma		0.85 (0.75-0.95)	**0.005**	0.83 (0.73-0.93)	**0.002**

Bold values indicate statistical significance (p < 0.05).

### Disparities After 2016 WHO Guidelines

A total of 719 glioma patients diagnosed in 2017 were identified; of which, 59.8% (n=430) had a reported 1p/19q codeletion test while 40.2% (n=289) did not. The median age was 45 years (IQR: 35-56) with 53.7% (n=386) being male. Non-Hispanic White, Hispanic White, Black, and other race/ethnicity patients had reported codeletion tests in 61.6%, 50.6%, 42.6%, and 72.5% of cases, respectively (p=0.008). Based on geographic region, a codeletion test was reported in 53.7%, 70.5%, 47.8%, and 68.9% of patients at Northeastern, Midwestern, Southern, and Western facilities, respectively (p=0.001). Patients with WHO grade III tumors (64.6%) were also more likely to have a reported codeletion test compared to WHO grade II tumors (56.5%) (p=0.030) (**Table 3**).

**Table 3:** Characteristics of patients with and without a reported 1p/19q codeletion test diagnosed in 2017. Frequencies and proportions are row-based.

Variable	Unreported 1p/19q test N = 289	Reported 1p/19q test N = 430	P-value
Age, median (IQR)	44 (35-55)	45 (36-56)	0.756
Sex			0.778
Male (n=386)	157 (40.7%)	229 (59.3%)	
Female (n=333)	132 (39.6%)	201 (60.4%)	
Race/Ethnicity			**0.008**
Non-Hispanic White (n=555)	213 (38.4%)	342 (61.6%)	
Hispanic White (n=77)	38 (49.4%)	39 (50.6%)	
Black (n=47)	27 (57.4%)	20 (42.6%)	
Other (n=40)	11 (27.5%)	29 (72.5%)	
Insurance			0.670
Private (n=452)	174 (38.5%)	278 (61.5%)	
Medicare (n=93)	39 (41.9%)	54 (58.1%)	
Medicaid/Other (n=132)	58 (43.9%)	74 (56.1%)	
Uninsured (n=42)	18 (42.9%)	24 (57.1%)	
Median household income in zip code			0.294
<$40,227 (n=96)	47 (49.0%)	49 (51.0%)	
$40,227-50,353 (n=135)	51 (37.8%)	84 (62.2%)	
$50,354-63,332 (n=185)	74 (40.0%)	111 (60.0%)	
≥$63,333 (n=303)	117 (38.6%)	186 (61.4%)	
Adults without high school degree in zip code			0.080
≥17.6% (n=134)	66 (49.3%)	68 (50.7%)	
10.9%-17.5% (n=176)	64 (36.4%)	112 (63.6%)	
6.3%-10.8% (n=202)	83 (41.1%)	119 (58.9%)	
<6.3% (n=207)	76 (36.7%)	131 (63.3%)	
Charlson-Deyo Comorbidity Score			0.382
0 (n=602)	242 (40.2%)	360 (59.8%)	
1 (n=83)	30 (36.1%)	53 (63.9%)	
2+ (n=34)	17 (50.0%)	17 (50.0%)	
Residential Region			0.565
Metropolitan (n=606)	248 (40.9%)	358 (59.1%)	
Urban (n=99)	35 (35.4%)	64 (64.6%)	
Rural (n=14)	6 (42.9%)	8 (57.1%)	
Geographic Region			**0.001**
Northeast (n=162)	75 (46.3%)	87 (53.7%)	
Midwest (n=122)	36 (29.5%)	86 (70.5%)	
South (n=69)	36 (52.2%)	33 (47.8%)	
West (n=106)	33 (31.1%)	73 (68.9%)	
Unknown (n=260)	109 (41.9%)	151 (58.1%)	
Facility Setting			0.330
Academic (n=245)	91 (37.1%)	154 (62.9%)	
Non-academic (n=214)	89 (41.6%)	125 (58.4%)	
Unknown (n=260)	109 (41.9%)	151 (58.1%)	
Distance travelled (miles), median (IQR)	13.9 (7.2-29.6)	16.8 (7.1-37.0)	0.387
1p/19q status			NA
Co-deleted	NA	380	
Not co-deleted	NA	50	
Histology			0.627
Oligodendroglioma (n=678)	274 (40.4%)	404 (59.6%)	
Oligoastrocytoma (n=41)	15 (36.6%)	26 (63.4%)	
WHO grade			**0.030**
2 (n=428)	186 (43.5%)	242 (56.5%)	
3 (n=291)	103 (35.4%)	188 (64.6%)	
Tumor location			0.697
Supratentorial (n=636)	254 (39.9%)	382 (60.1%)	
Infratentorial (n=0)	0	0	
NOS or other CNS (n=83)	35 (42.2%)	48 (57.8%)	
Tumor size			0.805
<5 cm (n=257)	96 (37.4%)	161 (62.6%)	
≥5 cm (n=263)	101 (38.4%)	162 (61.6%)	
Unknown	92 (46.2%)	107 (53.8%)	
Extent of resection			0.112
Biopsy only (n=65)	28 (43.1%)	37 (56.9%)	
STR (n=329)	144 (43.8%)	185 (56.2%)	
GTR (n=320)	115 (35.9%)	205 (64.1%)	
Unknown (n=5)	2 (40.0%)	3 (60.0%)	
Chemotherapy			0.187
Yes (n=471)	181 (38.4%)	290 (61.6%)	
No (n=222)	97 (43.7%)	125 (56.3%)	
Unknown (n=26)	11 (42.3%)	15 (57.7%)	
Radiotherapy			0.261
Yes (n=449)	173 (38.5%)	276 (61.5%)	
No (n=264)	113 (42.8%)	151 (57.2%)	
Unknown (n=6)	3 (50.0%)	3 (50.0%)	
Adjuvant treatment			0.522
Chemotherapy alone (n=59)	25 (42.4%)	34 (57.6%)	
Radiotherapy alone (n=32)	15 (46.9%)	17 (53.1%)	
Chemotherapy+Radiotherapy (n=412)	156 (37.9%)	256 (62.1%)	
Neither (n=186)	80 (43.0%)	106 (57.0%)	
Unknown (n=30)	13 (43.3%)	17 (56.7%)	

Bold values indicate statistical significance (p < 0.05).

NA, not applicable.

On multivariable analysis, Black race (OR 0.49; 95% CI 0.26-0.91; p=0.024) and reporting from hospitals in the Northeast (OR 0.50; 95% CI 0.30-0.84; p=0.009) and South (OR 0.42; 95% CI 0.22-0.78; p=0.007) compared to the Midwest were negatively associated with a reported 1p/19q codeletion test. Patients with WHO grade III tumors (OR 1.37; 95% CI 1.01-1.89; p=0.049) were significantly more likely to have a reported test (**Table 4**).

**Table 4:** Univariate and multivariable logistic regression evaluating factors associated with reporting of a 1p/19q codeletion test for patients diagnosed in 2017.

Variable	Reference	Univariate		Multivariable	
		OR (95% CI)	P-value	OR (95% CI)	P-value
Age	Continuous	1.00 (0.99-1.01)	0.756	–	–
Race/Ethnicity	Non-Hispanic White				
Hispanic White		0.64 (0.40-1.03)	0.067	0.68 (0.40-1.15)	0.149
Black		0.46 (0.25-0.84)	**0.012**	0.49 (0.26-0.91)	**0.024**
Other		1.64 (0.83-3.50)	0.174	1.66 (0.82-3.60)	0.174
Insurance	Private				
Medicare		0.87 (0.55-1.37)	0.536	–	–
Medicaid/Other		0.80 (0.54-1.18)	0.261	–	–
Uninsured		0.83 (0.44-1.60)	0.580	–	–
Residential region	Metropolitan				
Urban		1.27 (0.82-1.99)	0.295	–	–
Rural		0.92 (0.32-2.84)	0.884	–	–
Charlson-Deyo Comorbidity score	0				
1		1.19 (0.74-1.93)	0.479	–	–
2+		0.67 (0.33-1.35)	0.260	–	–
Adults without high school degree in zip code	<6.3%				
6.3%-10.8%		0.83 (0.56-1.24)	0.364	0.88 (0.57-1.34)	0.543
10.9%-17.5%		1.02 (0.67-1.54)	0.943	1.27 (0.76-2.12)	0.361
≥17.6%		0.60 (0.38-0.93)	**0.022**	0.86 (0.47-1.60)	0.641
Median household income in zip code	<$40,227				
$40,227-50,353		1.58 (0.93-2.69)	0.091	1.31 (0.74-2.33)	0.348
$50,354-63,332		1.44 (0.88-2.37)	0.151	1.16 (0.66-2.05)	0.605
≥$63,333		1.52 (0.96-2.42)	0.074	1.21 (0.66-2.23)	0.539
Geographic region	Midwest				
Northeast		0.49 (0.29-0.79)	**0.004**	0.50 (0.30-0.84)	**0.009**
South		0.38 (0.21-0.70)	**0.002**	0.42 (0.22-0.78)	**0.007**
West		0.93 (0.53-1.63)	0.790	0.92 (0.51-1.65)	0.776
Unknown		0.58 (0.36-0.91)	**0.020**	0.62 (0.38-0.99)	**0.047**
Facility setting	Academic				
Nonacademic		0.83 (0.57-1.21)	0.331	–	–
Unknown		0.82 (0.57-1.17)	0.273	–	–
WHO grade	Grade II				
Grade III		1.40 (1.03-1.91)	**0.031**	1.37 (1.01-1.89)	**0.049**
Histology	Oligodendroglioma				
Oligoastrocytoma		1.18 (0.62-2.31)	0.628	–	–

Bold values indicate statistical significance (p < 0.05).

### Disparities in 2017 Versus 2011-2015

Overall, patients were significantly more likely to have a reported 1p/19q codeletion test in 2017 versus 2011-2015 (OR 1.57; 95% CI 1.33-1.86; p<0.001). This trend was mirrored for most patient subgroups. However, Hispanic White patients (OR 1.28; 95% CI 0.77-2.11; p=0.341), Black patients (OR 1.04; 95% CI 0.54-1.94; p=0.915), rural residents (OR 1.20; 95% CI 0.33-4.46; p=0.783), and patients in the bottom quartile of household income (OR 1.31; 95% CI 0.85-2.03; p=0.228) were not statistically more likely to have a reported test in 2017 versus 2011-2015 (**Table 5**).

**Table 5:** Adjusted odds of a reported test for patients diagnosed in 2017 versus 2011-2015.

Variable	OR (95% CI)	P-value
Overall	1.57 (1.33-1.86)	**<0.001**
Race/Ethnicity		
Non-Hispanic White	1.61 (1.33-1.94)	**<0.001**
Hispanic White	1.28 (0.77-2.11)	0.341
Black	1.04 (0.54-1.94)	0.915
Other	3.26 (1.57-7.22)	**0.002**
Insurance		
Private	1.54 (1.25-1.90)	**<0.001**
Medicare	1.84 (1.18-2.91)	**0.007**
Medicaid/Other	1.46 (1.00-2.16)	0.054
Uninsured	1.80 (0.93-3.53)	0.082
Residential region		
Metropolitan	1.51 (1.26-1.81)	**<0.001**
Urban	2.22 (1.42-3.53)	**<0.001**
Rural	1.20 (0.33-4.46)	0.783
Adults without high school degree in zip code		
<6.3%	1.56 (1.15-2.12)	**0.004**
6.3%-10.8%	1.32 (0.97-1.79)	0.081
10.9%-17.5%	2.07 (1.48-2.91)	**<0.001**
≥17.6%	1.44 (0.99-2.09)	0.058
Median household income in zip code		
<$40,227	1.31 (0.85-2.03)	0.228
$40,227-50,353	1.93 (1.33-2.83)	**<0.001**
$50,354-63,332	1.59 (1.15-2.22)	**0.006**
≥$63,333	1.61 (1.25-2.08)	**<0.001**
Geographic region		
Northeast	1.58 (1.12-2.23)	**0.009**
Midwest	2.05 (1.34-3.20)	**0.001**
South	1.42 (0.84-2.38)	0.187
West	1.61 (1.03-2.56)	**0.042**
Facility setting		
Academic	1.86 (1.39-2.50)	**<0.001**
Nonacademic	1.47 (1.08-1.99)	**0.013**
WHO grade		
Grade II	1.34 (1.08-1.66)	**0.007**
Grade III	2.03 (1.56-2.66)	**<0.001**
Histology		
Oligodendroglioma	1.54 (1.30-1.83)	**<0.001**
Oligoastrocytoma	2.39 (1.25-4.76)	**0.009**

Bold values indicate statistical significance (p < 0.05).

### 1p/19q Codeletion Testing and Adjuvant Treatment

On univariate analysis, reported testing was associated with an increased likelihood of receiving adjuvant treatment (OR 1.35; 95% CI 1.20-1.51; p<0.001). An interaction analysis identified GTR as a significant confounding variable. After adjusting for GTR and other relevant confounders, a reported 1p/19q codeletion test was found to be independently associated with increased odds of receiving adjuvant treatment (OR 1.73; 95% CI 1.46-2.04; p<0.001). The interaction between a reported codeletion test and GTR was found to be significantly associated with decreased odds of receiving adjuvant treatment (OR 0.63; 95% CI 0.49-0.82; p<0.001) (**Table 6**).

**Table 6:** Univariate and multivariable logistic regression evaluating factors associated with receipt of adjuvant treatment.

Variable	Reference	Univariate		Multivariable	
		OR (95% CI)	P-value	OR (95% CI)	P-value
1p/19q testing	Unreported test				
Reported test		1.35 (1.20-1.51)	**<0.001**	1.73 (1.46-2.04)	**<0.001**
GTR	No				
Yes		0.62 (0.55-0.69)	**<0.001**	0.63 (0.49-0.82)	**<0.001**
1p/19q testing * GTR	Unreported test or no GTR				
Reported test and GTR		–	–		
Year of Diagnosis	2011-2015				
2017		1.65 (1.38-1.97)	**<0.001**	2.04 (1.67-2.51)	**<0.001**
Age	Continuous	1.02 (1.02-1.02)	**<0.001**	0.99 (0.98-1.00)	0.113
Race	Non-Hispanic White				
Hispanic White		0.87 (0.71-1.08)	0.205	0.95 (0.74-1.23)	0.706
Black		0.79 (0.63-1.07)	0.054	0.92 (0.70-1.21)	0.555
Other		0.95 (0.74-1.22)	0.678	0.97 (0.73-1.30)	0.846
Insurance	Private				
Medicare		1.04 (0.88-1.23)	0.666	0.68 (0.54-0.86)	**0.001**
Medicaid/Other		1.09 (0.93-1.28)	0.288	1.09 (0.90-1.31)	0.396
Uninsured		0.74 (0.60-0.91)	**0.004**	0.80 (0.63-1.03)	0.080
Residential region	Metropolitan				
Urban		0.96 (0.82-1.13)	0.635	–	**-**
Rural		1.48 (0.94-2.43)	0.104	–	**-**
Charlson-Deyo Comorbidity score	0				
1		1.03 (0.87-1.23)	0.726	–	**-**
2+		1.06 (0.83-1.37)	0.639	–	**-**
Adults without high school degree in zip code	<6.3%				
6.3%-10.8%		0.96 (0.83-1.11)	0.572	0.90 (0.75-1.07)	0.237
10.9%-17.5%		0.98 (0.84-1.14)	0.769	1.03 (0.83-1.27)	0.810
≥17.6%		0.78 (0.66-0.91)	**0.002**	0.79 (0.62-1.02)	0.067
Median household income in zip code	<$40,227				
$40,227-50,353		1.24 (1.03-1.50)	**0.020**	1.24 (0.99-1.54)	0.059
$50,354-63,332		1.19 (0.99-1.42)	0.062	1.03 (0.82-1.30)	0.768
≥$63,333		1.15 (0.97-1.35)	0.102	0.97 (0.76-1.24)	0.809
Geographic region	Midwest				
Northeast		0.73 (0.61-0.89)	**0.002**	0.70 (0.56-0.87)	**0.001**
South		0.48 (0.38-0.61)	**<0.001**	0.48 (0.36-0.62)	**<0.001**
West		0.74 (0.59-0.92)	**0.007**	0.68 (0.53-0.87)	**0.002**
Unknown		0.38 (0.32-0.46)	**<0.001**	0.34 (0.26-0.44)	**<0.001**
Facility setting*	Academic				
Nonacademic		1.24 (1.06-1.44)	**0.006**	–	**-**
Unknown		0.56 (0.49-0.64)	**<0.001**	–	**-**
WHO grade	II				
III		6.36 (5.55-7.29)	**<0.001**	7.17 (6.20-8.30)	**<0.001**
Histology	Oligodendroglioma				
Oligoastrocytoma		1.20 (1.07-1.36)	**0.003**	1.22 (1.06-1.41)	**0.006**

*Facility setting was not included in the multivariable model due to collinearity with geographic region.

Bold values indicate statistical significance (p < 0.05).

## Discussion

In an analysis of a national oncology registry, we evaluated disparities in reported testing for 1p/19q codeletion in patients with a histological diagnosis of either oligodendroglioma or oligoastrocytoma given the marker’s inclusion as a mandatory component of diagnosis in the 2016 WHO classification of CNS tumor guidelines. A 14.0% increase in reported testing was identified from 2011 to 2017. However, the rise was not equitable among all patient subgroups. While disparities among uninsured patients and those in zip codes with lower educational attainment dissipated from 2011-2015 to 2017, geographic regional disparities were maintained. Black patients were also found to have an insignificant change in testing rates from 2011-2015 to 2017. Subsequently, they were also found to have disproportionately lower odds of testing following the new WHO guidelines. The likelihood of receiving adjuvant treatment was also found to be independently associated with reported codeletion testing status.

Although the precise mechanisms behind these disparities could not be ascertained in our analysis, these are likely multifactorial in nature. Laboratory capabilities such as fluorescence *in situ* hybridization, most commonly used to detect codeletion, may not be available onsite at hospitals with smaller case volumes (19). Inequities in access to high-volume facilities among Black, Hispanic, and lower socioeconomic status patients undergoing brain tumor craniotomy have been extensively documented (8, 10, 20). Heterogeneous geographic disparities in care and a lower likelihood of travelling large distances to high-volume hospitals, previous observations among these patient populations, may compound financial and/or logistical barriers to centralized neuro-oncological care (21–23). Socioeconomic status is often intertwined with race/ethnicity and has also been shown to influence outcomes and access to treatments for glioma patients (9, 11, 24). In our analysis, the Northeast and South were highlighted as geographic regions in the United States that should garner additional focus for addressing disparities in testing. Additionally, the insignificant improvement in testing rates among Black patients was especially concerning given the increased likelihood of testing for most other demographic subgroups. Future studies are needed to more specifically identify how these disparities arise in order to develop targeted initiatives that promote more equitable access to care.

There remains conflicting evidence with regard to the impact of race/ethnicity on OS among oligodendroglioma and oligoastrocytoma patients. Analyses from the Central Brain Tumor Registry of the United States (CBRTUS) and Surveillance, Epidemiology, and End Results (SEER) registry have previously noted a lower incidence of primary oligodendroglial tumors among Black patients, but similar or higher OS, compared to White patients (25, 26). In contrast, Shin et al. evaluated anaplastic oligodendroglioma patients in the NCDB and found that Black patients had significantly lower OS compared to non-Black patients, even after only selecting patients who received chemoradiotherapy (27). However, molecular profile was not evaluated in any of these studies. While it has been suggested that patient race may predispose gliomas to molecular profiles that are associated with discrepancies in survival, the causes of the molecular heterogeneity of gliomas remain poorly understood (28, 29). More likely, the aforementioned inequities in access to care, such as for molecular testing, play a substantial role in influencing differences in patient management and survival.

Studies on the epidemiology of oligodendroglioma diagnoses and testing for O-6-Methylguanine-DNA Methyltransferase (MGMT) promoter methylation in glioblastoma (GBM) patients may provide further insights into disparities in molecular testing rates. An analysis of the CBTRUS identified that the incidence of oligodendroglioma significantly declined from 2000-2013 for White and Asian/Pacific Islander patients, but not Black patients (26). The increasing emphasis on using 1p/19q codeletion status for diagnosis during this time period, including recommendations for testing in tumors with an oligodendroglial component from the National Comprehensive Cancer Network, suggest that this epidemiological variation may be due to differences in utilization of molecular diagnostics between races (30). In addition, Lamba et al. performed an analysis of the NCDB from 2010-2016 and found that GBM patients of lower socioeconomic status, including insurance and median household income, were disproportionately less likely to be tested for MGMT methylation status. However, patient race/ethnicity was not identified as a significant predictor. MGMT-tested patients were also more likely to receive chemotherapy compared to untested patients, which is comparable to our findings on adjuvant treatment for oligodendroglioma/oligoastrocytoma patients (31). As a potential consequence of inequitable molecular testing, racial, socioeconomic, and geographic disparities may exacerbate pre-existing barriers to clinical trial enrollment since molecular profile has become an increasingly emphasized criterion for screening patients with primary CNS tumors (32–34).

A primary concern regarding disparities in testing rates is the potential for inferior patient outcomes, especially for heterogeneously managed tumors like oligodendrogliomas and oligoastrocytomas. Our analysis indicated that having a reported 1p/19q codeletion test was independently associated with receiving adjuvant treatment. In the literature, differences in patient survival based on adjuvant management of these tumors have been previously noted. Two randomized clinical trials published in 2013 demonstrated an enhanced response to radiotherapy with the addition of adjuvant procarbazine, lomustine, and vincristine (PCV) in anaplastic oligodendroglioma patients with a 1p/19q codeletion compared to those without the mutation (3, 4). Additionally, while we await the results from the ongoing CODEL trial comparing temozolomide with radiotherapy and PCV with radiotherapy for codeleted WHO grade III oligodendrogliomas, results from the initial study design demonstrated superior progression-free survival for patients receiving temozolomide with radiotherapy as compared to temozolomide alone (35). In our study cohort, it is likely that patients without a reported test are comprised of both 1p/19q intact and codeleted patients. Given the impact of reported testing rates on adjuvant management and the influence of molecular profile on tumor treatment response, disparities in molecular testing may influence outcomes for oligodendroglioma and oligoastrocytoma patients.

There are some limitations to this study. The retrospective study design may subject the cohort to selection bias. Variables included in the analysis were limited to those available in the database. Notably, IDH status is not collected in NCDB (14). Although 1p/19q codeletions predominately co-occur with IDH mutations, the most complete evaluation of the 2016 WHO guidelines would include both markers. The analytical technique and timing of codeletion testing was also lacking, limiting our interpretation on optimal integration of testing into clinical practice. Furthermore, the extent of missing data on facility setting and region, since these variables are suppressed for patients aged <40, should be considered when evaluating the results. The potential for miscoding of ICD-O-3 codes should also be acknowledged. Given that the NCDB only captures patients from CoC-accredited hospitals, the study cohort was not population-based. In addition, our analysis is contextualized as evaluating the reported testing rates for codeletion in the NCDB, rather than the actual testing rate. The NCDB PUF acknowledges the likelihood that case coverage of site-specific factors, including 1p/19q codeletion, may be limited in the database (14). This may be due to factors including data availability at the time of abstraction. However, other site-specific factors, like WHO grade, are extensively coded throughout the study period.

## Conclusion

Routine molecular profiling of histological oligodendrogliomas and oligoastrocytomas serves as an opportunity to more accurately classify these tumors, better inform prognosis, and optimize patient management. Despite the 2016 WHO guidelines, disparities in facility geographic region persisted and new disparities in race/ethnicity were identified for reported 1p/19q codeletion testing. Since the likelihood of receiving adjuvant treatment was found to be associated with reported testing, these disparities may further influence patient outcomes. These findings highlight the need for more targeted research efforts to identify mechanisms behind these disparities as well as initiatives to promote more equitable access to testing.

## Data Availability Statement

The data analyzed in this study is subject to the following licenses/restrictions: NCDB PUFs are only available through an application process to investigators associated with CoC-accredited cancer programs. Requests to access these datasets should be directed to https://www.facs.org/quality-programs/cancer/ncdb/puf.

## Ethics Statement

Ethical review and approval was not required for the study on human participants in accordance with the local legislation and institutional requirements. Written informed consent for participation was not required for this study in accordance with the national legislation and the institutional requirements.

## Author Contributions

All authors listed have made a substantial, direct, and intellectual contribution to the work and approved it for publication.

## Funding

Funding for this study was received from K12 CA90628 (SK).

## Conflict of Interest

The authors declare that the research was conducted in the absence of any commercial or financial relationships that could be construed as a potential conflict of interest.

## Publisher’s Note

All claims expressed in this article are solely those of the authors and do not necessarily represent those of their affiliated organizations, or those of the publisher, the editors and the reviewers. Any product that may be evaluated in this article, or claim that may be made by its manufacturer, is not guaranteed or endorsed by the publisher.
