# Supplementary figures and images for: Disparities in Reported Testing for 1p/19q Codeletion in Oligodendroglioma and Oligoastrocytoma Patients: An Analysis of the National Cancer Database
Source: Front Oncol. 2021 Nov 9;11:746844. doi: 10.3389/fonc.2021.746844 (PMC8630738; doi:10.3389/fonc.2021.746844)

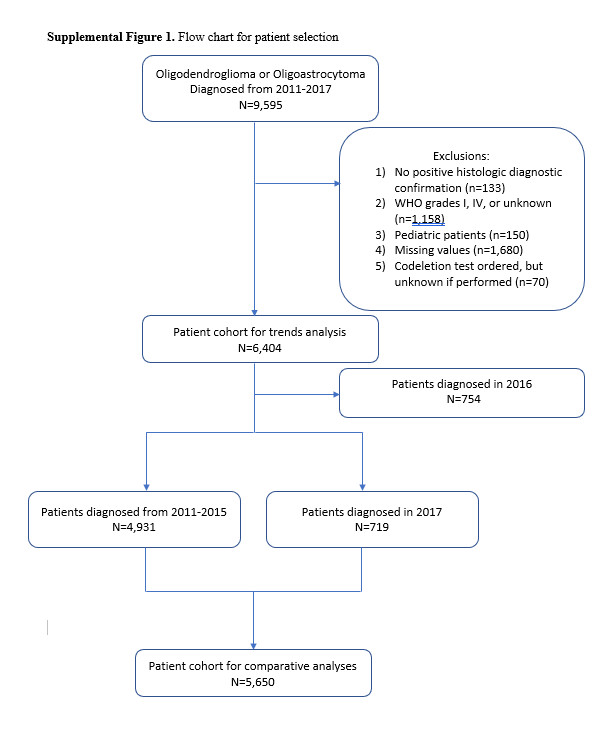

Supplement: Supplementary file 1 [file Image_1.jpeg]
