# Supplementary material for: Disparities in Reported Testing for 1p/19q Codeletion in Oligodendroglioma and Oligoastrocytoma Patients: An Analysis of the National Cancer Database
Source: Front Oncol. 2021 Nov 9;11:746844. doi: 10.3389/fonc.2021.746844 (PMC8630738; doi:10.3389/fonc.2021.746844)
Supplement: Supplementary file 2 [file Table_1.docx]

**Supplemental Table 1.** Definitions of covariates according to the NCDB participant user file

| Covariate | Definition |
| --- | --- |
| Charlson-Deyo comorbidity score | A weighted comorbidity index mapped from International Classification of Diseases (ICD) codes for 15 different comorbid conditions. |
| Household income | The median household income for each patient’s zip code of residence. Categorized into quartiles. |
| Educational attainment | The percentage of adults age ≥25 years in a patient’s zip code who did not graduate from high school, according to the American Community Survey data. Categorized into quartiles. |
| Residential region | Categorized as metropolitan, urban, or rural according to the US Department of Agriculture Economic Research Service |
| Facility setting | Categorized as either academic, non-academic, or unknown according to the classification of its structural characteristics by the CoC Accreditation program |
| Distance travelled | The distance, in miles, between the patient’s zip code centroid and the hospital that reported the case |
| Tumor location | Classified as supratentorial, infratentorial, or not otherwise specified (NOS) or other CNS according to the ICD-O-3 topography code for the primary site |
| Tumor size | The largest dimension of the diameter of the solid primary tumor and categorized as <5 cm, ≥5 cm, or unknown |
| Extent of resection | Categorized into biopsy only, local excision or subtotal resection (STR), gross total resection (GTR), or unknown according to the American College of Surgeons CoC Facility Oncology Registry Data System manual |
| Adjuvant treatment | Defined as receipt of chemotherapy and radiotherapy, chemotherapy alone, radiotherapy alone, or neither chemotherapy nor radiotherapy |

**Supplemental Table 2.** Characteristics of patients with and without a reported 1p/19q codeletion test diagnosed from 2011-2015. Frequencies and proportions are column-based.

| Variable | Unreported 1p/19q test  N = 2,582 | Reported 1p/19q test  N = 2,349 | Total  N = 4,931 | P-value |
| --- | --- | --- | --- | --- |
| Age, median (IQR) | 44 (34-56) | 44 (33-54) | 44 (34-55) | **0.004** |
| Sex |  |  |  | 0.256 |
| Male | 1427 (55.3%) | 1336 (56.9%) | 2763 (56.0%) |  |
| Female | 1155 (44.7%) | 1013 (43.1%) | 2168 (44.0%) |  |
| Race/Ethnicity |  |  |  | **0.034** |
| Non-Hispanic White | 2075 (80.4%) | 1958 (83.4%) | 4033 (81.8%) |  |
| Hispanic White | 196 (7.6%) | 160 (6.8%) | 356 (7.2%) |  |
| Black | 166 (6.4%) | 114 (4.9%) | 280 (5.7%) |  |
| Other | 145 (5.6%) | 117 (5.0%) | 262 (5.3%) |  |
| Insurance |  |  |  | **<0.001** |
| Private | 1635 (63.3%) | 1598 (68.0%) | 3233 (65.6%) |  |
| Medicare | 347 (13.4%) | 262 (11.2%) | 609 (12.4%) |  |
| Medicaid/Other | 376 (14.6%) | 337 (14.3%) | 713 (14.5%) |  |
| Uninsured | 224 (8.7%) | 152 (6.5%) | 376 (7.6%) |  |
| Median household income in zip code |  |  |  | **0.010** |
| <$40,227 | 455 (17.6%) | 342 (14.6%) | 797 (16.2%) |  |
| $40,227-50,353 | 553 (21.4%) | 480 (20.4%) | 1033 (20.9%) |  |
| $50,354-63,332 | 609 (23.6%) | 573 (24.4%) | 1182 (24.0%) |  |
| ≥$63,333 | 965 (37.4%) | 954 (40.6%) | 1919 (38.9%) |  |
| Adults without high school degree in zip code |  |  |  | **<0.001** |
| ≥17.6% | 584 (22.6%) | 388 (16.5%) | 972 (19.7%) |  |
| 10.9%-17.5% | 641 (24.8%) | 536 (22.8%) | 1177 (23.9%) |  |
| 6.3%-10.8% | 709 (27.5%) | 725 (30.9%) | 1434 (29.1%) |  |
| <6.3% | 648 (25.1%) | 700 (29.8%) | 1348 (27.3%) |  |
| Charlson-Deyo Comorbidity Score |  |  |  | 0.552 |
| 0 | 2140 (82.9%) | 1952 (83.1%) | 4092 (83.0%) |  |
| 1 | 307 (11.9%) | 262 (11.2%) | 569 (11.5%) |  |
| 2+ | 135 (5.2%) | 135 (5.7%) | 270 (5.5%) |  |
| Residential Region |  |  |  | 0.472 |
| Metropolitan | 2183 (84.5%) | 1976 (84.1%) | 4159 (84.3%) |  |
| Urban | 364 (14.1%) | 331 (14.1%) | 695 (14.1%) |  |
| Rural | 35 (1.4%) | 42 (1.8%) | 77 (1.6%) |  |
| Geographic Region |  |  |  | **<0.001** |
| Northeast | 653 (25.3%) | 470 (20.0%) | 1123 (22.8%) |  |
| Midwest | 396 (15.3%) | 436 (18.6%) | 832 (16.9%) |  |
| South | 282 (10.9%) | 169 (7.2%) | 451 (9.1%) |  |
| West | 270 (10.5%) | 334 (14.2%) | 604 (12.2%) |  |
| Unknown | 981 (38.0%) | 940 (40.0%) | 1921 (39.0%) |  |
| Facility Setting |  |  |  | 0.113 |
| Academic | 902 (34.9%) | 832 (35.4%) | 1734 (35.2%) |  |
| Non-academic | 699 (27.1%) | 577 (24.6%) | 1276 (25.9%) |  |
| Unknown |  |  |  |  |
| Distance travelled (miles), median (IQR) | 14.8 (6.2-36.4) | 17.5 (7.2-42.9) | 16 (6.7-39.2) | 0.368 |
| 1p/19q status |  |  |  | NA |
| Co-deleted | NA | 1464 (62.3%) | NA |  |
| Not co-deleted | NA | 885 (37.7%) | NA |  |
| Histology |  |  |  | **0.005** |
| Oligodendroglioma | 1657 (64.2%) | 1596 (67.9%) | 3253 (66.0%) |  |
| Oligoastrocytoma | 925 (35.8%) | 753 (32.1%) | 1678 (34.0%) |  |
| WHO grade |  |  |  | 0.224 |
| 2 | 1476 (57.2%) | 1383 (58.9%) | 2859 (58.0%) |  |
| 3 | 1106 (42.8%) | 966 (41.1%) | 2072 (42.0%) |  |
| Tumor location |  |  |  | **0.033** |
| Supratentorial | 2446 (94.7%) | 2261 (96.3%) | 4707 (95.5%) |  |
| Infratentorial | 43 (1.7%) | 25 (1.1%) | 68 (1.4%) |  |
| NOS or other CNS | 93 (3.6%) | 63 (2.7%) | 156 (3.2%) |  |
| Tumor size |  |  |  | **0.002** |
| <5 cm | 996 (38.6%) | 938 (39.9%) | 1934 (39.2%) |  |
| ≥5 cm | 848 (32.8%) | 842 (35.8%) | 1690 (34.3%) |  |
| Unknown | 738 (28.6%) | 569 (24.2%) | 1307 (26.5%) |  |
| Extent of resection |  |  |  | **<0.001** |
| Biopsy only | 305 (11.8%) | 193 (8.2%) | 498 (10.1%) |  |
| STR | 1309 (50.7%) | 1162 (49.5%) | 2471 (50.1%) |  |
| GTR | 946 (36.6%) | 983 (41.8%) | 1929 (39.1%) |  |
| Unknown | 22 (0.9%) | 11 (0.5%) | 33 (0.7%) |  |
| Chemotherapy |  |  |  | **<0.001** |
| Yes | 1182 (45.8%) | 1277 (54.4%) | 2459 (49.9%) |  |
| No | 1277 (49.5%) | 1003 (42.7%) | 2280 (46.2%) |  |
| Unknown | 123 (4.8%) | 69 (2.9%) | 192 (3.9%) |  |
| Radiotherapy |  |  |  | 0.070 |
| Yes | 1218 (47.2%) | 1171 (49.9%) | 2389 (48.4%) |  |
| No | 1335 (51.7%) | 1157 (49.3%) | 2492 (50.5%) |  |
| Unknown | 29 (1.1%) | 21 (0.9%) | 50 (1.0%) |  |
| Adjuvant treatment |  |  |  | **<0.001** |
| Chemotherapy alone | 244 (9.5%) | 334 (14.2%) | 578 (11.7%) |  |
| Radiotherapy alone | 260 (10.1%) | 216 (9.2%) | 476 (9.7%) |  |
| Chemotherapy+Radiotherapy | 936 (36.3%) | 938 (39.9%) | 1874 (38.0%) |  |
| Neither | 1005 (38.9%) | 778 (33.1%) | 1783 (36.2%) |  |
| Unknown | 137 (5.3%) | 83 (3.5%) | 220 (4.5%) |  |

**Supplemental Table 3.** Characteristics of patients with and without a reported 1p/19q codeletion test diagnosed in 2017. Frequencies and proportions are column-based.

| Variable | Unreported 1p/19q test  N = 289 | Reported 1p/19q test  N = 430 | Total  N = 719 | P-value |
| --- | --- | --- | --- | --- |
| Age, median (IQR) | 44 (35-55) | 45 (36-56) | 45 (35-56) | 0.756 |
| Sex |  |  |  | 0.778 |
| Male | 157 (54.3%) | 229 (53.3%) | 386 (53.7%) |  |
| Female | 132 (45.7%) | 201 (46.7%) | 333 (46.3%) |  |
| Race/Ethnicity |  |  |  | **0.008** |
| Non-Hispanic White | 213 (73.7%) | 342 (79.5%) | 555 (77.2%) |  |
| Hispanic White | 38 (13.1%) | 39 (9.1%) | 77 (10.7%) |  |
| Black | 27 (9.3%) | 20 (4.7%) | 47 (6.5%) |  |
| Other | 11 (3.8%) | 29 (6.7%) | 40 (5.6%) |  |
| Insurance |  |  |  | 0.670 |
| Private | 174 (60.2%) | 278 (64.7%) | 452 (62.9%) |  |
| Medicare | 39 (13.5%) | 54 (12.6%) | 93 (12.9%) |  |
| Medicaid/Other | 58 (20.1%) | 74 (17.2%) | 132 (18.4%) |  |
| Uninsured | 18 (6.2%) | 24 (5.6%) | 42 (5.8%) |  |
| Median household income in zip code |  |  |  | 0.294 |
| <$40,227 | 47 (16.3%) | 49 (11.4%) | 96 (13.4%) |  |
| $40,227-50,353 | 51 (17.6%) | 84 (19.5%) | 135 (18.8%) |  |
| $50,354-63,332 | 74 (25.6%) | 111 (25.8%) | 185 (25.7%) |  |
| ≥$63,333 | 117 (40.5%) | 186 (43.3%) | 303 (42.1%) |  |
| Adults without high school degree in zip code |  |  |  | 0.080 |
| ≥17.6% | 66 (22.8%) | 68 (15.8%) | 134 (18.6%) |  |
| 10.9%-17.5% | 64 (22.1%) | 112 (26.0%) | 176 (24.5%) |  |
| 6.3%-10.8% | 83 (28.7%) | 119 (27.7%) | 202 (28.1%) |  |
| <6.3% | 76 (26.3%) | 131 (30.5%) | 207 (28.8%) |  |
| Charlson-Deyo Comorbidity Score |  |  |  | 0.382 |
| 0 | 242 (83.7%) | 360 (83.7%) | 602 (83.7%) |  |
| 1 | 30 (10.4%) | 53 (12.3%) | 83 (11.5%) |  |
| 2+ | 17 (5.9%) | 17 (4.0%) | 34 (4.7%) |  |
| Residential Region |  |  |  | 0.565 |
| Metropolitan | 248 (85.8%) | 358 (83.3%) | 606 (84.3%) |  |
| Urban | 35 (12.1%) | 64 (14.9%) | 99 (13.8%) |  |
| Rural | 6 (2.1%) | 8 (1.9%) | 14 (1.9%) |  |
| Geographic Region |  |  |  | **0.001** |
| Northeast | 75 (26.0%) | 87 (20.2%) | 162 (22.5%) |  |
| Midwest | 36 (12.5%) | 86 (20.0%) | 122 (17.0%) |  |
| South | 36 (12.5%) | 33 (7.7%) | 69 (9.6%) |  |
| West | 33 (11.4%) | 73 (17.0%) | 106 (14.7%) |  |
| Unknown | 109 (37.7%) | 151 (35.1%) | 260 (36.2%) |  |
| Facility Setting |  |  |  | 0.330 |
| Academic | 91 (31.5%) | 154 (35.8%) | 245 (34.1%) |  |
| Non-academic | 89 (30.8%) | 125 (29.1%) | 214 (29.8%) |  |
| Unknown | 109 (37.7%) | 151 (35.1%) | 260 (36.2%) |  |
| Distance travelled (miles), median (IQR) | 13.9 (7.2-29.6) | 16.8 (7.1-37.0) | 15.1 (7.2-35.5) | 0.387 |
| 1p/19q status |  |  |  | NA |
| Co-deleted | NA | 380 (88.4%) | NA |  |
| Not co-deleted | NA | 50 (11.6%) | NA |  |
| Histology |  |  |  | 0.627 |
| Oligodendroglioma | 274 (94.8%) | 404 (94.0%) | 678 (94.3%) |  |
| Oligoastrocytoma | 15 (5.2%) | 26 (6.0%) | 41 (5.7%) |  |
| WHO grade |  |  |  | **0.030** |
| 2 | 186 (64.4%) | 242 (56.3%) | 428 (59.5%) |  |
| 3 | 103 (35.6%) | 188 (43.7%) | 291 (40.5%) |  |
| Tumor location |  |  |  | 0.697 |
| Supratentorial | 254 (87.9%) | 382 (88.8%) | 636 (88.5%) |  |
| Infratentorial | 0 (0%) | 0 (0%) | 0 (0%) |  |
| NOS or other CNS | 35 (12.1%) | 48 (11.2%) | 83 (11.5%) |  |
| Tumor size |  |  |  | 0.805 |
| <5 cm | 96 (33.2%) | 161 (37.4%) | 257 (35.7%) |  |
| ≥5 cm | 101 (34.9%) | 162 (37.7%) | 263 (36.6%) |  |
| Unknown | 92 (31.8%) | 107 (24.9%) | 199 (27.7%) |  |
| Extent of resection |  |  |  | 0.112 |
| Biopsy only | 28 (9.7%) | 37 (8.6%) | 65 (9.0%) |  |
| STR | 144 (49.8%) | 185 (43.0%) | 329 (45.8%) |  |
| GTR | 115 (39.8%) | 205 (47.7%) | 320 (44.5%) |  |
| Unknown | 2 (0.7%) | 3 (0.7%) | 5 (0.7%) |  |
| Chemotherapy |  |  |  | 0.187 |
| Yes | 181 (62.6%) | 290 (67.4%) | 471 (65.5%) |  |
| No | 97 (33.6%) | 125 (29.1%) | 222 (30.9%) |  |
| Unknown | 11 (3.8%) | 15 (3.5%) | 26 (3.6%) |  |
| Radiotherapy |  |  |  | 0.261 |
| Yes | 173 (59.9%) | 276 (64.2%) | 449 (62.4%) |  |
| No | 113 (39.1%) | 151 (35.1%) | 264 (36.7%) |  |
| Unknown | 92 (31.8%) | 107 (24.9%) | 199 (27.7%) |  |
| Adjuvant treatment |  |  |  | 0.522 |
| Chemotherapy alone | 25 (8.7%) | 34 (7.9%) | 59 (8.2%) |  |
| Radiotherapy alone | 15 (5.2%) | 17 (4.0%) | 32 (4.5%) |  |
| Chemotherapy+Radiotherapy | 156 (54.0%) | 256 (59.5%) | 412 (57.3%) |  |
| Neither | 80 (27.7%) | 106 (24.7%) | 186 (25.9%) |  |
| Unknown | 13 (4.5%) | 17 (4.0%) | 30 (4.2%) |  |
